# Stabilization of liquid crystal blue phases by carbon nanoparticles of varying dimensionality†

**DOI:** 10.1039/d0na00276c

**Published:** 2020-05-01

**Authors:** Adam P. Draude, Tejas Y. Kalavalapalli, Maria Iliut, Ben McConnell, Ingo Dierking

**Affiliations:** Department of Physics and Astronomy, University of Manchester Manchester M13 9PL UK ingo.dierking@manchester.ac.uk; Department of Chemical Engineering, Faculty of Applied Science, Delft University of Technology Building 58, Van der Maasweg 9 Delft 2629 HZ The Netherlands; Department of Materials, University of Manchester Manchester M13 9PL UK

## Abstract

The thermal stabilization of blue phases is a subject that has been of scientific and technological interest since their discovery. Meanwhile, carbonaceous nanomaterials such as C60 fullerenes, carbon nanotubes and graphene have generated interdisciplinary interest spanning across solid-state physics, organic chemistry, colloids, all the way to soft matter physics. Herein, the stabilization of liquid crystal blue phases by doping with C60, single-walled carbon nanotubes and graphene oxide is described. All three types of particles are found to extend the combined temperature range of blue phases I and II by a factor of ∼5. Furthermore, mixtures of pairs of different materials, and all three types are shown to stabilize the blue phases. The temperature range of the blue phases is shown to grow at the expense of the cholesteric phase. This leads to a blue phase-cholesteric-smecticA phase triple-point in all cases except that of doping with carbon nanotubes. The mechanisms of this thermal stabilization are discussed in light of theoretical descriptions for other established systems.

## Abstract 2

Carbon nanomaterials of differing dimensionality, namely fullerenes, nanotubes and graphene oxide are shown to stabilize the Blue Phases at the expense of the N* phase until its complete disappearance. A BP–N*–SmA* triple point is observed.

## Graphical abstract

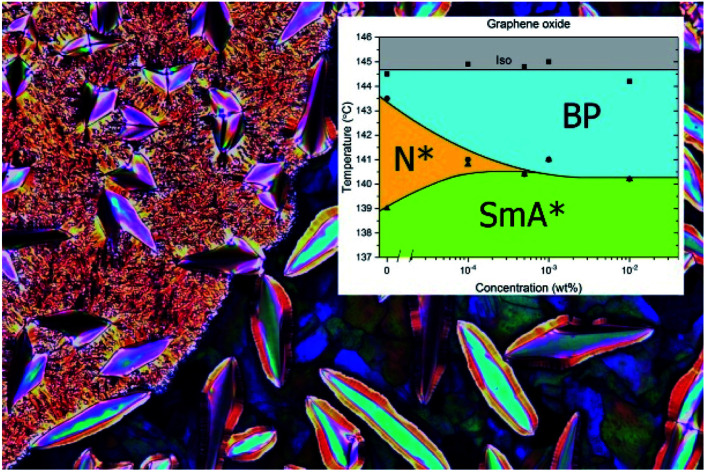

## Introduction

**Fig. 1:**
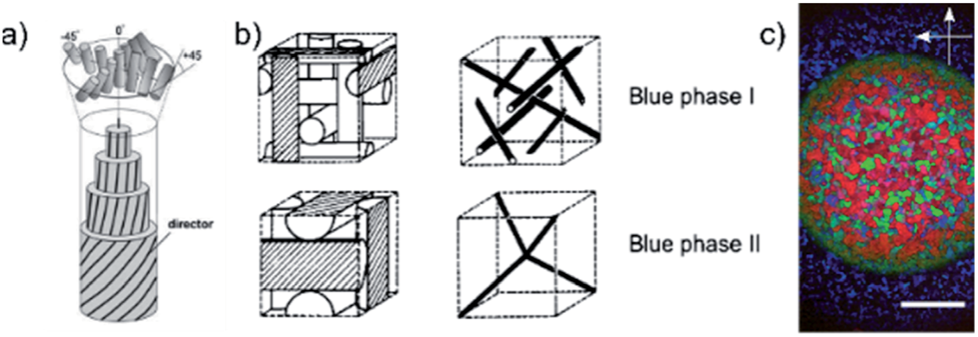
(a) Schematic structures of the double-twist cylinders formed by the liquid crystal molecules in the BPI and BPII phases (b) the corresponding unit cells and the structure of disclinations where the double twist cylinders meet. The BPIII phase is locally cubic but has no long-ranged ordering *i.e.* it is amorphous. (c) Polarizing optical microscopy image of the compound CE8. There is a radial temperature gradient across the observation hole of the hotstage, with the center being cooler than the edges. Thus, the outer ring texture is that of BPII and the inner circle is BPI. The BPIII cannot be seen in the photograph, as it appears as a very dark blue fog only. The scale bar is 300 μm. (a) and (b) Reproduced from Kikuchi^39^ with permission Springer Nature.

Of the many liquid crystal phases which exist between the three dimensionally ordered crystal state and the isotropic liquid, the frustrated phases are possibly the most enigmatic. We will here only discuss thermotropic liquid crystalline materials, those that are exhibited by change of temperature, while lyotropic phases have not been investigated within the context of this work. This leaves us mainly with two groups of frustrated phases (i) the blue phases,^1,2^ which are thermodynamically located between the isotropic and the chiral nematic (N*), or cholesteric, phase and (ii) the twist grain boundary phases (TGB),^3,4^ which appear between the chiral nematic and the fluid smectic phases (SmA*, SmC*). Both classes of frustrated phases result from a competition between chirality, thus the formation of a helical superstructure, and thermodynamics. In the case of the Blue Phases (BP) the material would like to preserve an isotropic character, while at the same time forming a helical superstructure. The result is a frustrated phase consisting of double twist cylinders mediated by a cubic structure of disclination lines (Fig. 1(a)).

As these defects cost elastic energy, the temperature regime of the blue phase is generally very small, of the order of 1 K. In the past it was thus taken for granted that there were no reasonable applications of these phases. They were regarded of purely academic interest until a method was introduced to widen the BP temperature regime through polymer stabilization.^5–7^ This opened the gates for display applications of BPs through light modulation *via* the Kerr effect. Such displays, which were built to prototype level by Samsung,^8,9^ were much faster in optical response than standard nematic LCDs, with refresh rates up to 250 Hz, excellent viewing characteristics, and most of all the lack of a need for alignment layers, which is a considerable cost factor in production and a source of detrimental dust particles. Nevertheless, polymer stabilization only appeared to work with certain polymer mixtures under very specific conditions. There is thus still the need for a robust methodology of the expansion of the BP temperature regime and a number of different methods were suggested^2^ with more or less success. The addition of bent-core molecular dopants was shown to widen the blue phase,^10^ as was the addition of colloidal particles.^11–13^ Also combinations of different methods had beneficial effects, although not quite being additive.^10^ A promising approach, not too dissimilar to polymer stabilization, is the additive mixing of a polymer into the BP, which was shown to be increasingly effective the shorter the added polymer was made.^14^ In fact, extrapolation to a molecular weight equivalent to a dimer, gave a temperature regime of the blue phase, equivalent to what had been observed for some liquid crystal dimers, ∼50 K.^15^

Materials based upon the two-dimensional material graphene have also been investigated as stabilizing additives to the blue phases. Several approaches have used graphene oxide functionalized with either flexible alkyl chains,^16,17^ CoPt nanoparticles,^18^ aminoazobenzene groups,^19,20^ or mesogenic units^21^ in an effort to increase the chemical compatibility with thermotropic liquid crystals at high concentrations of doping. In all cases, the total temperature range of the blue phases was expanded by approximately 2 K or less. Some reports further suggest that as the concentration of the dopant is increased further, the temperature range of the blue phases actually decreases.^16,20,21^ Furthermore, the concentrations of graphene material that caused the maximal blue phase ranges were rather large, of the order 0.1 wt% or higher.

As mentioned above, also at the transition from cholesteric to fluid smectic, frustrated phases can be observed, the twist grain boundary phases.^22,23^ These exhibit a discontinuous helical superstructure, which can be commensurate or incommensurate,^24^ with adjacent smectic blocks being rotated with respect to each other and separated by grain boundaries of regular arrays of screw dislocations (Fig. 1(b)). Also here those defects costs energy, being responsible for the generally narrow temperature regime of the TGB phases. Whilst this paper focuses on the blue phases, experiments equivalent to those for BP were also carried out for the TGB phase where possible. In this paper, we predominantly present results of a systematic study of the influence of carbon nanoparticles of varying dimensionality and concentration on the phase stability of the blue phases. These are namely fullerenes as 0D, nanotubes as 1D and graphene oxide as 2D carbon materials. Hereon, the collective term blue phase is used to describe BPI-III collectively. We show that the blue phase is stabilized at the expense of the cholesteric phase, and report the observation of a BP–N*–SmA* triple point. We further present results for the mixtures of combinations of two respective particles, as well as the mixture of all three materials. Results are discussed and interpreted considering model structures of phases and dopants.

## Experimental

**Fig. 2:**
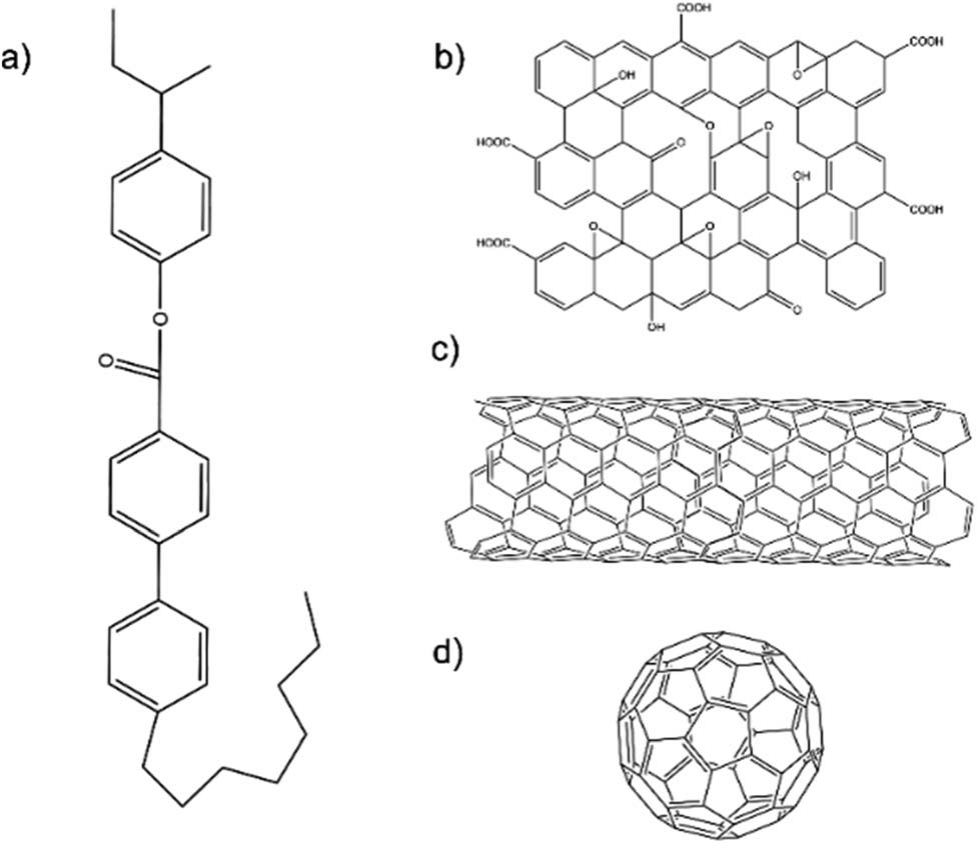
The molecular structures of the liquid crystal compound used in this investigation, CE8 (a). The example schematic structures of the three carbon nanomaterials used are also shown: (b) graphene oxide, (c) single-walled carbon nanotube and (d) C60 fullerene. Note these are not to scale.

The liquid crystal used in this study was CE8. Schematic diagrams of the chemical structures of CE8 and each of the nanomaterials used in the investigations are shown in Fig. 2. C60 fullerenes and SWNT were obtained from Snaucke Elements and Carbolex Inc., respectively, both in powder form. The graphene oxide (GO) used in this study was synthesized by a modified Hummers' method for a previous study^25^ and had since been dispersed in dimethylformamide (DMF) and stored in a refrigerator at 5 °C to mitigate thermal reduction.

**Fig. 3:**
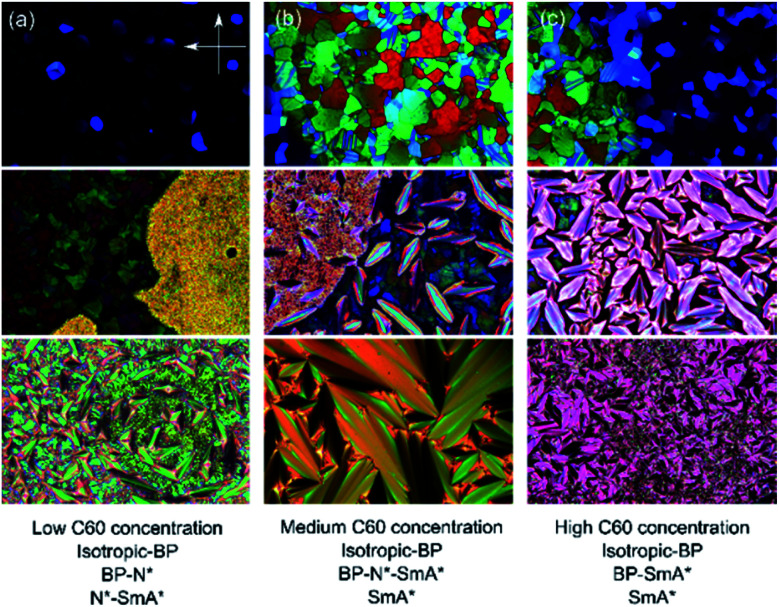
Illustrative POM textures showing the effect of (a) low, (b) medium, and (c) high fullerene concentration and temperature (vertical) on the phase behavior of CE8. The BP–N*–SmA* triple point texture can be seen in the central texture micrograph. The orientations of the polarizer and analyzer are the same in all the images.

To dope the nanomaterials into the LCs, the general procedure was to disperse the nanomaterial in a co-solvent of the LC, mix both fluids and evaporate the solvent at high temperature over several hours. To ensure all the co-solvent was evaporated, an equivalent amount of the LC and solvent with no nanomaterial was mixed and left to evaporate under the same conditions for the same amount of time. Since the clearing point of liquid crystals is very sensitive to the presence of solvents, the un-doped sample's transition temperatures were observed to be different from those of the pure LC if any solvent still remained. For the case of SWNTs and GO with CE8 the co-solvent used was DMF (Fisher). Toluene (Sigma Aldrich) was used as the solvent for C60, yielding the distinctive purple-colored solution for which C60 is known.^26^ The nanoparticle-doped LCs were then placed between two glass coverslips. In the case of the nanotubes and the graphene oxide, the dispersion of nanomaterial in the LC was heterogeneous, with small aggregates visible in polarized microscopy (Fig. S2 and S3†). On the length scales available to optical microscopy, the fullerenes appeared to disperse homogeneously (Fig. 3). Transition temperatures were measured by the observation of the textures of the LC in polarized optical microscopy (POM) whilst the temperature was changed using a Linkam LTSE350 hotstage and TP94 temperature controller with a precision of 0.1 K. The temperature was changed at a rate of 0.1 °C min^−1^. The blue phase of CE8 appeared to be more pronounced in polarized microscopy on cooling from the isotropic phase. Hence, all samples were measured on cooling.

## Results and discussion

The addition of C60, SWCNTs or GO all increased the width of the blue phases. The isotropic–BP transition and the temperature at which the SmA* phase appeared remained relatively unchanged as larger amounts of nanomaterial were added with the exception of added GO. In this case, the N*–SmA* transition raised slightly in temperature by ∼1 K. The chiral nematic phase decreased in width to accommodate the stabilized blue phases. For the cases of fullerene and GO doping, this reduction in width of the N* phase lead to a BP–N*–SmA* triple point at medium concentrations (Fig. 3(b), middle image).

**Fig. 4:**
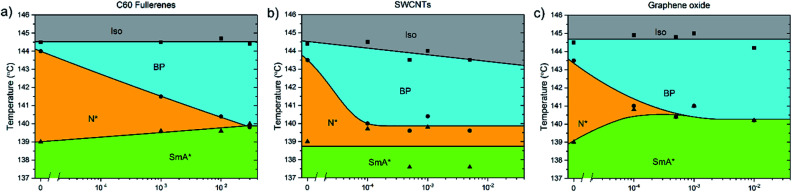
Phase diagrams showing the dependence of the high temperature phases of CE8 on (a) C60, (b) SWCNT and (c) graphene oxide concentration. The uncertainty of the temperature measurements is of the order of the data points, ∼0.1 K. The solid lines are a guide to the eye.

The addition of graphene oxide sheets to CE8 to rid the mixture of the N* phase to only observe a BP–SmA* phase sequence is seemingly more efficient than adding fullerenes, since it takes more than ten times the concentration by weight of fullerenes to completely form the BP phase at the expense of the N* phase (Fig. 4). In both cases, the temperature width of blue phases ceases to increase further with the addition of nanomaterials after the BP–N*–SmA* triple point is observed. The SmA* phase is observed approximately 1 K higher in temperature as compared to the pure compound. In the case of the SWCNTs, the N* phase never completely disappears at any concentration of SWCNTs tested and the BP width saturates at around 4 K. At even larger concentrations of nanotubes, agglomeration leads to a slight variability in the observed width of the N* phase, and no further trend is observed. Here, the transition temperature to SmA* does not appear to change with increasing nanotube content.

**Fig. 5:**
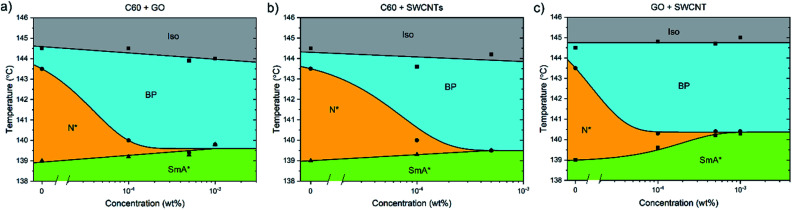
Phase diagrams of CE8 with added pairs of nanoparticle types in the weight ratio 1 : 1. The concentrations are expressed as the total nanoparticle content by weight %. The uncertainty of the temperature measurements is of the order of the data points, ∼0.1 K. The solid lines are a guide to the eye.

Pairs of different nanomaterials were added in a weight ratio of 1 : 1 (Fig. 5). When either graphene oxide and fullerenes or fullerenes and nanotubes are added together to CE8, the effect is similar to that observed when solely graphene oxide is added, with the triple point being reached at approximately 5 × 10^−4^ wt% total nanoparticle content. When nanotubes and GO are combined in CE8, the BP is not stabilized further than 10^−4^ wt% nanoparticle content but, contrary to the GO only case, the N* phase persists for higher concentrations. In these systems the N*–SmA* transition is raised in temperature about 1 K leading to a triple-point at 10^−3^ wt%. From the experiments using each nanomaterial in isolation, this slight rise in the temperature of the N*–SmA* transition can be attributed to the influence of the GO sheets. Graphene oxide sheets are thought to have clusters of sp^2^-bonded domains on their surfaces. Upon such domains, CE8 molecules would tend to align parallel on the surface due to π–π stacking interactions and thus act as nucleation sites for the SmA* phase.

**Fig. 6:**
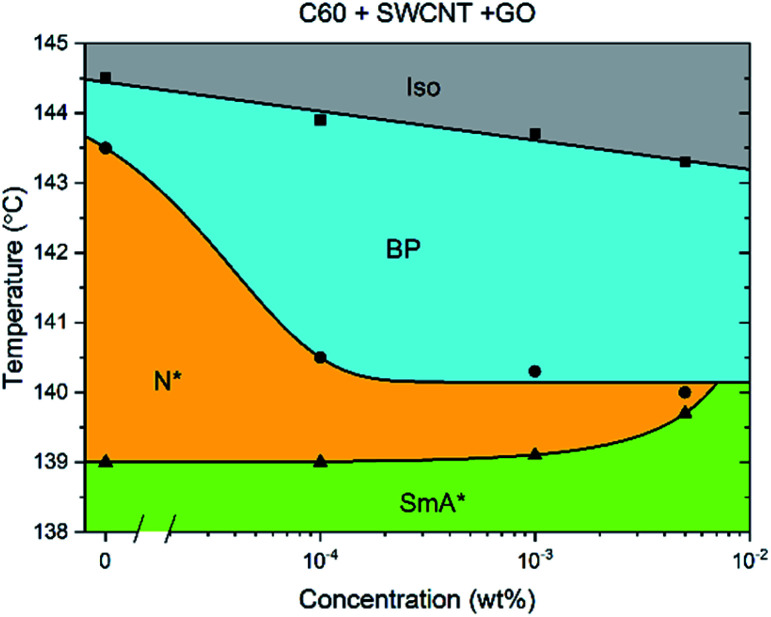
Phase diagram of CE8 with C60, GO and SWCNT all added together in a 1 : 1 : 1 ratio. The concentration is expressed as the total nanoparticle content by weight %. The uncertainty of the temperature measurements is of the order of the data points, ∼0.1 K. The solid lines are a guide to the eye.

When C60, GO and SWCNTs are added in a 1 : 1 : 1 ratio to CE8 (Fig. 6), again the BP width does not increase much past a concentration of 10^−4^ wt%, indicating that the three individual mechanisms of stabilization all occur, and that their contributions are likely to be roughly additive as far as the data suggest.

The mechanism of stabilization is expected to be different for the different nanomaterials, since each have very different sizes and aspect ratios. Previously, it has been shown that polymer stabilization of blue phases occurs due to a decrease in the free energy associated with defects and disclinations in the lattice of double-twist cylinders which constitute BPI and BPII. The polymer fibers form preferentially in the defect sites and disclination lines to minimize this free energy and hence stabilize the phase.^5,27^ It is also expected that a similar mechanism is behind reports of other nanoparticles,^12,28^ increasing the blue phase temperature range by 2–5 K. Furthermore, even dispersing oligomers of varying chain length has shown to stabilize the blue phases.^14^ Accompanying modeling to these studies^14,15^ indicates that the maximum temperature range achievable occurs at a chain length of a dimer. Fullerenes are of a similar size, though their shape is more like that of a colloidal quantum dot. C60 has previously been added to blue phases by Khoo *et al.*,^29^ however this study reported on the photorefractive effects of the composite and the phase diagram was not studied in detail.

**Fig. 7:**
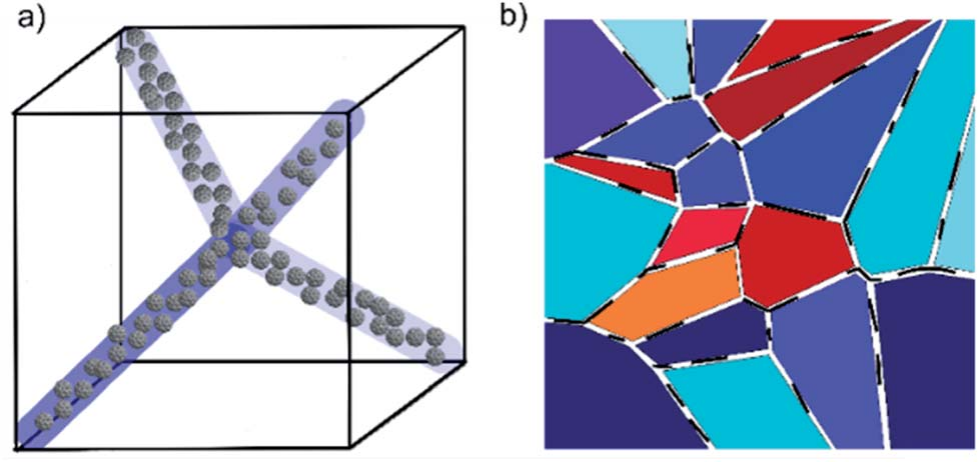
Schematic diagrams of blue phase stabilization by (a) C60 fullerenes and (b) graphene oxide sheets. In the former case, bunches of fullerenes are shown to fill the regions in and around the disclination lines. The case for BPII is shown, but the same principle can be extended to the more complex BPI. In the case of graphene oxide stabilization, the disruptive presence of the sheets (black lines) forces grain boundaries in the structures which give rise to the platelet textures of BPI and BPII, but it is proposed that these cost less energy than they usually would without GO and hence stabilize the phase.

The widths of the disclination lines in BPI and BPII are estimated to be 10 nm,^30^ which is larger than the diameter of a single fullerene, ∼10 Å.^26^ It is therefore probable that several fullerenes collect in the defects in a disorderly fashion as they are formed out of the isotropic melt (Fig. 7(a)), rather than forming simple linear chains as has been proposed in illustrations of quantum dots in blue phases in some studies.^12,31^ Recently, Gharbi *et al.*^32^ have shown that gold nanoparticles doped into blue phases can spontaneously and reversibly rearrange themselves to best accommodate the different defect lattice structures of BPI and BPII. They propose a model where particles preferentially aggregate at the meetings of dislocation lines, so it is possible that in the present system the largest concentration of C60 is found at the same points in the BP lattices.

The widths of SWCNTs are of the same order as a fullerene, but their lengths are polydisperse and up to the range of microns, much larger than the size of the BP unit cell (∼300 nm). Furthermore, strong van der Waals and π–π stacking interactions between nanotubes causes them to form bundles, the diameters of which have been measured *via* scanning electron microscopy (SEM) to be approximately 60 nm.^33^ These bundles are larger than the widths of disclination lines in the blue phase and are therefore less effective in reducing the free energy density of the liquid crystal phase. The fullerenes on the other hand can adapt their packing within the disclination lines as to maximise the reduction in free energy. It is however not completely clear what the mechanism is behind the stabilizing effect of small amounts of nanotubes. On the one hand the elastic deformation energy of the defects and disclination lines are largely reduced, while on the other hand the double-twist cylinder bulk is elastically deformed. This indicates that the maximal thermal stabilization given by the nanotubes should in fact be smaller than that of the fullerenes, which is experimentally observed. Recently, Kemiklioglu and Chien^34^ reported the effect of the addition of carbon nanotubes to a polymer-stabilized blue phase system. In contrast to our findings, the total width of the blue phases was seen to decrease with increasing nanotube content. This effect could be due to the fact that in the polymer-stabilized system, the disclinations of the blue phases are already filled by the polymer network, and then the addition of further solid material simply leads to elastic deformation of the larger double-twist structure and an increase in the total free energy of the system, which may destabilize the phase.

Unless the sample is cooled from the isotropic phase or BPIII phase slowly in the presence of alignment layers on the substrates,^35^ or *via* polymer-stabilization in an electric field,^36^ the cubic blue phases form with multiple domains and grain boundaries (Fig. 3). The grain boundaries, like the lattice of defects, cost energy. Being of monolayer thickness, graphene oxide sheets sitting at the grain boundaries could lower the overall free energy and thus stabilize the phase (Fig. 7(b)). Within the scope of this study, it has not been possible to clarify if the GO does indeed collect at these boundaries. However with a mean size of 4.4 μm the sheets are certainly too large (Fig. S1†) to form inclusions in the lattice of disclinations, as smaller particles can. Moreover, it has been shown that GO collects at other interfaces, such as that between two immiscible liquids.^37,38^ It is thus reasonable to assume that the stabilizing effect of the graphene oxide stems from a reduction of the free energy due to the platelet grain boundaries by a collection of GO at these interfaces.

The TGB phase of the widely-available liquid crystal cholesteryl pelargonate was also briefly investigated to see if carbonaceous nanomaterials could also stabilize this frustrated phase. However, the only nanomaterial for which a suitable co-solvent could be found to aid the dispersion process was C60 (toluene). Furthermore, it was found that on cooling and heating, the presence of fullerenes at up to 0.1 wt% had no effect on the width of the TGB phase. A possible explanation for this is that the adjacent phases, the N* and SmA* phases, are much more viscous than the isotropic and blue phases, meaning the fullerenes may be unable to diffuse to, and efficiently fill, the high-energy cores of the screw dislocations that are present in TGB phases.

## Conclusions

In this work the influence of carbonaceous nanomaterials of different dimensionality, namely 0D fullerenes, 1D nanotubes and 2D graphene oxide sheets, on the stability of liquid crystal blue phases was investigated. Such a study is motivated through the possibility of an easily accessible material for novel display and electro-optic applications with enhanced performance at lower cost than conventional liquid crystals. It was found that small amounts of all of these additives have a positive effect on the thermal stabilization of the blue phases, in some cases leading to a complete vanishing of the cholesteric phase to maximize the blue phase temperature range. Whilst for the 0D case (fullerenes) the effect can be explained in terms of established theoretical models, the use of larger particles like nanotubes and 2D materials for the purposes of stabilizing blue phases do not fit with the idea of filling the lattice of defect lines present in BPI and BPII.

## Conflicts of interest

There are no conflicts to declare.

## Supplementary Material

NA-002-D0NA00276C-s001
