# Supplementary material for: Stabilization of liquid crystal blue phases by carbon nanoparticles of varying dimensionality
Source: Nanoscale Adv. 2020 May 1;2(6):2404–9. doi: 10.1039/d0na00276c (PMC9419272; doi:10.1039/d0na00276c)
Supplement: NA-002-D0NA00276C-s001 [file NA-002-D0NA00276C-s001.pdf]

Electronic Supplementary Material (ESI) for Nanoscale Advances.
This journal is © The Royal Society of Chemistry 2020

Electronic Supporting Information

Stabilization of liquid crystal blue phases by carbon nanoparticles of varying dimensionality

Adam Paul Draude, Tejas Yangnavalk Kalavalpalli, Maria Iliut, Ben McConnell, Ingo Dierking

1. Size distribution of graphene oxide

The size distribution of the graphene oxide used to dope the CE8 (as described in the main text) was measured by spin coating a dilute suspension of GO in DMF onto a Si-290 wafer and then imaging the flakes using scanning electron microscopy (SEM) in secondary electron mode. The flakes are not circular, and so an equivalent diameter was defined by measuring each flakes length horizontally through the center. Since the spin coating should not prescribe any preferred orientation to the flakes, this method gives a statistical measure of the flake size. 259 flakes were measured to form the distribution (Figure S3). The distribution matches well that of a log-normal distribution (shown). The arithmetic mean equivalent diameter was 4.4µm.

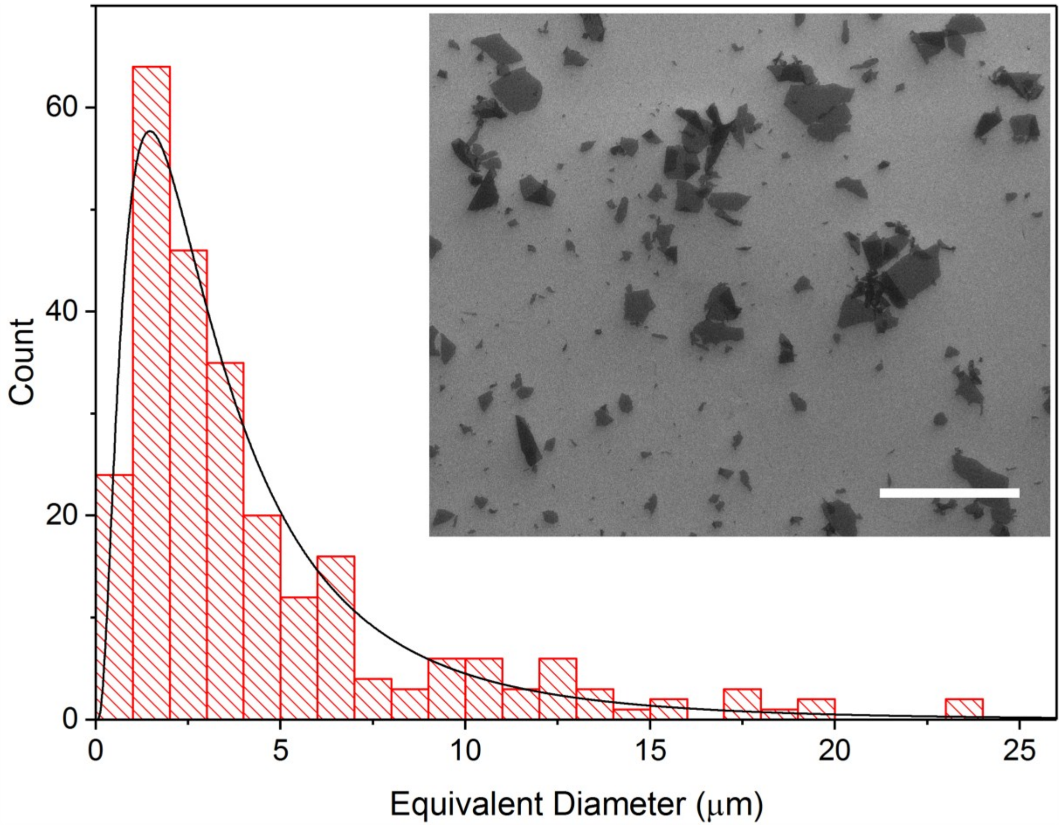

Figure S1. The measured GO flake size distribution from 259 flakes using scanning electron micrographs such as the example shown in the inset. The scale bar is 50µm.

2. Aggregation of graphene oxide and nanotubes in CE8

Whilst C60 fullerenes did not appear to phase separate from CE8 at any concentration tested, graphene oxide and carbon nanotubes did aggregate to some extent even at relatively small concentrations. An example for the case

of GO at a concentration of $5x10^{-4}$wt% is given in Figure S2. When the concentration is increased further, larger dark regions appear which cover a large area. An example for the case of SWCNTs at a concentration of 10^{-3}wt% is shown in Figure S3.

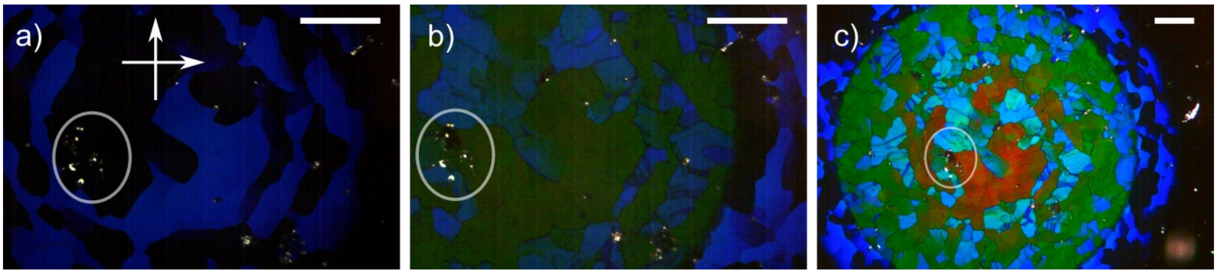

Figure S2. Aggregation of graphene oxide sheets in CE8. The concentration is $5x10^{-4}$wt%. Aggregates of GO are visible in crossed polarizers as either bright or dark spots depending on their orientation. To illustrate this, the same aggregate is circled in this temperature series as the sample cools from 144.8°C-144.7°C (a-c). The scale bars are all 100µm. The orientations of the polarizer and analyzer are the same for all the images.

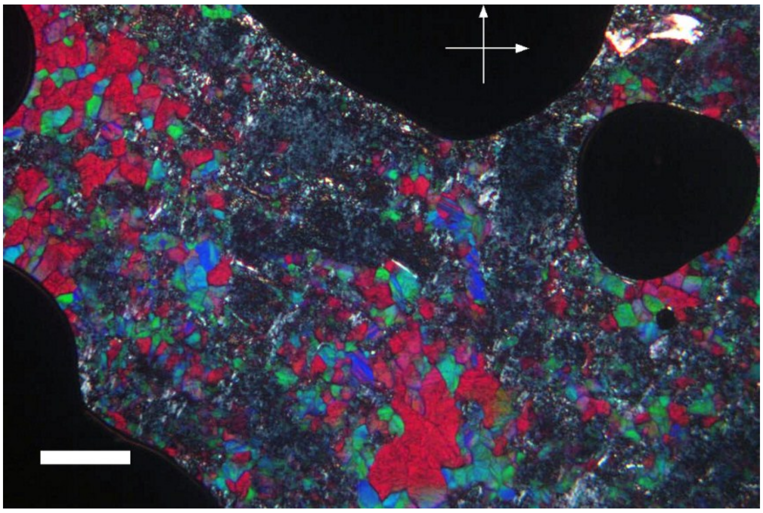

Figure S3. At a high concentration of 10^{-3}wt%, SWCNTs bundle into large grey/black areas. The pitch black parts of the image are air bubbles. The scale bar is 100µm.
